# Reactive astrocytes with reduced function of glutamate transporters in the *App^NL‐G‐F^* knock‐in mice

**DOI:** 10.1002/alz.085633

**Published:** 2025-01-03

**Authors:** Raúl Loera‐Valencia, Ipsit Srivastava, Julen Goikolea, Tamer Ayberk Kaya, Maria Latorre Leal, Marta Pereira Iglesias, Laura Alvarez‐Jimenez, Luis Enrique Arroyo‐García, Makoto Shimozawa, Per Nilsson, André Fisahn, Maria Lindskog, Silvia Maioli

**Affiliations:** ^1^ Karolinska Institutet, Stockholm Sweden; ^2^ ITESM, Chihuahua, CI Mexico; ^3^ Karolinska Institutet, Stockholm, Stockholm Sweden; ^4^ Division of Neurogeriatrics, Center for Alzheimer Research, Department of Neurobiology, Care Sciences and Society (NVS), Karolinska Institutet, Stockholm Sweden; ^5^ Karolinska Institutet, Stockholm, Solna Sweden; ^6^ Karolinska Institutet, Dept NVS, Div of Neurogeriatrics, 171 64 Solna, Solna Sweden; ^7^ Division of Neurogeriatrics, Karolinska Institutet, Solna Sweden; ^8^ Uppsala Universitet, Uppsala, Uppsala Sweden

## Abstract

**Background:**

Alzheimer’s disease (AD) is associated with synaptic and memory dysfunction. A pathological hallmark of the disease is reactive astrogliosis, with reactive astrocytes surrounding amyloid plaques in the brain. Astrocytes have also been shown to be actively involved in disease progression, nevertheless, mechanistic information about their role in synaptic transmission during AD pathology is lacking. Astrocytes maintain synaptic transmission by taking up extracellular glutamate during synaptic activity through astrocytic glutamate transporter GLT‐1, but its function has been difficult to measure in real‐time in AD pathology.

**Method:**

In this study, we used an *App* knock‐in AD model (*App^NL‐G‐F^*) carrying the Swedish, Arctic and Beyreuther mutations associated with AD and exhibiting AD‐like Aβ plaque deposition and memory impairment. Using immunohistochemistry, patch‐clamp of astrocytes, and western blot from tissue and FACS isolated astrocytes.

**Result:**

We found that *App^NL‐G‐F^* mice at 6‐8 months of age have astrocytes with clearly altered morphology compared to wild‐type (WT). Moreover, astrocyte glutamate clearance function in *App^NL‐G‐F^* mice, measured as electrophysiological recordings of glutamate transporter currents, was severely impaired compared to WT animals. We suggest that the loss of function of GLT‐1 is not related to altered protein levels since no changes in GLT‐1 levels in synaptosomes nor FACS‐isolated astrocytes from *App^NL‐G‐F^* mice were found. This phenotype can potentially alter synaptic transmission and/or contribute to glutamate excitotoxicity.

**Conclusion:**

Our data suggest that astrocytic glutamate transporters are affected by excess Aβ42 in the brain contributing to synaptic dysfunction in the hippocampus. Thus, our data indicate that restoring astrocyte synaptic function could be a potential therapeutic strategy to treat AD.

